# On the validity of fMRI mega-analyses using data processed with different pipelines

**DOI:** 10.1162/imag_a_00522

**Published:** 2025-04-28

**Authors:** Elodie Germani, Xavier Rolland, Pierre Maurel, Camille Maumet

**Affiliations:** Univ Rennes, Inria, CNRS, Inserm, Rennes, France

**Keywords:** neuroimaging, analytical variability, pipelines, validity, data re-use

## Abstract

In neuroimaging and functional magnetic resonance imaging (fMRI), many derived data are made openly available in public databases. These can be re-used to increase sample sizes in studies and thus, improve robustness. In fMRI studies, raw data are first preprocessed using a given analysis pipeline to obtain subject-level contrast maps, which are then combined into a group analysis. Typically, the subject-level analysis pipeline is identical for all participants. However, derived data shared on public databases often come from different workflows, which can lead to different results. Here, we investigate how this analytical variability, if not accounted for, can induce false positive detections in mega-analyses combining subject-level contrast maps processed with different pipelines. We use the Human Connectome Project (HCP) multi-pipeline dataset, containing contrast maps for N = 1,080 participants of the HCP Young-Adult dataset, whose raw data were processed and analyzed with 24 different pipelines. We performed between-groups analyses with contrast maps from different pipelines in each group and estimated the rates of pipeline-induced detections. We show that, if not accounted for, analytical variability can lead to inflated false positive rates in studies combining data from different pipelines.

## 1 Introduction

Over the past few years, concerns have been raised regarding the lack of reproducibility of neuroimaging findings (Botvinik-Nezer & Wager, 2023;Button et al., 2013;Poldrack et al., 2017). In particular, the low statistical power of studies was criticized, as effectively leading to low probabilities of identifying true effects but also to high probabilities of reporting false positive findings in the literature (Button et al., 2013). Researchers proposed different approaches to increase sample sizes, and thus statistical power, for instance with the development of large-scale studies (Sudlow et al., 2015;Van Essen et al., 2013). However, acquiring such an amount of data is costly, and due to the challenge of finding participants, these studies often contain a few number of data per participant. In functional magnetic resonance imaging (fMRI) – a brain imaging technique used to study brain activity under different conditions – these datasets often only cover a limited subset of brain functions. This effectively restricts the flexibility of the research questions that may be explored. A potential solution to increase sample size while avoiding these challenges is to re-use the data already acquired in other studies into meta- or mega-analyses (Salimi-Khorshidi et al., 2009).

With the increased adoption of open science practices (Niso et al., 2022;Poldrack & Gorgolewski, 2014;Poline et al., 2012) and the development of dedicated research infrastructures (Barillot et al., 2016;Gorgolewski et al., 2015;Markiewicz et al., 2021), such as NeuroVault (Gorgolewski et al., 2015) and OpenNeuro (Markiewicz et al., 2021), more and more neuroimaging data from various studies have been made available to the scientific community. This includes raw data at the subject level, which can be re-analyzed using the same processing steps and combined in a mega-analysis, but also derived data (i.e., already processed) at the subject or group level. At the group level, derived data can be used in meta-analyses to build consensus results across multiple studies (Salimi-Khorshidi et al., 2009), but there are several limitations to this method due to publication bias (Ioannidis et al., 2014).

At the subject level, individual contrast maps (after the subject-level processing) from different studies can be combined using mega-analyses (also known as*individual patient data (IPD) meta-analysis*). Their re-use is more optimal than raw data, not only because sharing of statistic maps is easier due to reduced privacy requirements, but also because it avoids having to perform costly re-computations. Indeed, fMRI studies require multiple processing steps on the data, both at the subject level (preprocessing of the raw fMRI data to prepare them for statistical analysis, and first-level analysis for each participant) and at the group level (second-level statistical analysis using the subject-level contrast maps resulting from first-level analysis). However, it is unlikely that derived data available on public databases come from the same pipeline. In addition, derived fMRI datasets can come from adaptable pipelines that apply different processing steps depending on which data are available (see, for example, fMRIprep;Esteban et al., 2019). In practice, in those mega-analyses, confounds (such as differing pipelines) are typically accounted for by adding a nuisance covariate to the model (Alfaro-Almagro et al., 2021). This approach is useful to remove any detections that are induced by those confounds, yet when it comes to the pipeline, it is sometimes not straightforward to identify which aspect of the pipeline constitutes a nuisance factor and should be modeled.

Multiple studies have shown that different implementations of a processing pipeline can lead to different results in neuroimaging. These changes can arise from different levels of variations: different software packages (Bowring et al., 2019) or software packages version (Gronenschild et al., 2012), different algorithms and processing steps (Botvinik-Nezer et al., 2020;Li et al., 2021;S. C. Strother, 2006), different software environment (Glatard et al., 2015), etc. InBotvinik-Nezer et al. (2020), 70 teams analyzed the same task-fMRI dataset, each with their usual pipeline, leading to 70 different analytical conditions. They found substantial differences in the results obtained across teams, in terms of statistic maps but also answers to binary hypotheses. This variability resulting from the processing and analysis protocol used on the data is also known as*analytical variability*.

Here, we systematically investigate how analytical variability impacts the results of fMRI mega-analyses with data from different pipelines (when no additional measures are taken to account for that variability). Previous studies have focused on how analytical variability affects the reproducibility of existing results in neuroimaging, by using different pipelines to complete a similar analysis in which the processing applied on all subject data is the same and comparing the results obtained across pipelines using different processing pipelines. In addition, dedicated frameworks for optimizing the choice of pipelines have been proposed based on an estimation of reproducibility performance (LaConte et al., 2003;S. Strother et al., 2004). Notably, solutions to use different subject-level processing pipelines have been suggested in this context (Churchill et al., 2015).

We explore the impact of pipeline-based differences on the results of between-group analyses that compare populations whose data were processed differently at the subject level. While, currently, this is not common practice, this setup could be useful in order to compare healthy versus pathological populations using processed data from different datasets (for example, data of healthy participants from the minimally processed dataset of the Human Connectome Project (HCP) compared with patient data from another dataset). We carry out a series of between-groups analyses, with each group corresponding to subject-level contrast maps randomly sampled from the Human Connectome Project (HCP) Young Adult dataset (Van Essen et al., 2013) and processed with different pipelines (one pipeline in each group). Since participants in all groups are sampled from the same dataset, we expect no population-based differences, and, therefore, all observed differences are attributable to the differing pipelines.

## 2 Material and Methods

The goal of this study is to test the validity of between-group analyses using subject-level contrast maps processed with different pipelines (when differences in pipelines are not accounted for in the statistical model). In the following sections, the term “pipeline” is used to refer to the subject-level pipeline.

The steps performed to estimate this validity are presented inFigure 1. First, we randomly sampled subject-level contrast maps processed through different pipelines from the HCP multi-pipeline dataset (Germani et al., 2023) (seeSection 2.1). Then, for each pair of pipelines, we performed a between-group analysis (seeSection 2.2). This group comparison was repeated 1,000 times in order to estimate the empirical rate of (pipeline-induced) significant differences. In the following, we denote this as the “false positive rate,” considering that those may be equivalently seen as false detections in a between-group analysis using a simple statistical model in which differences in pipelines are not accounted for.

**Fig. 1.**
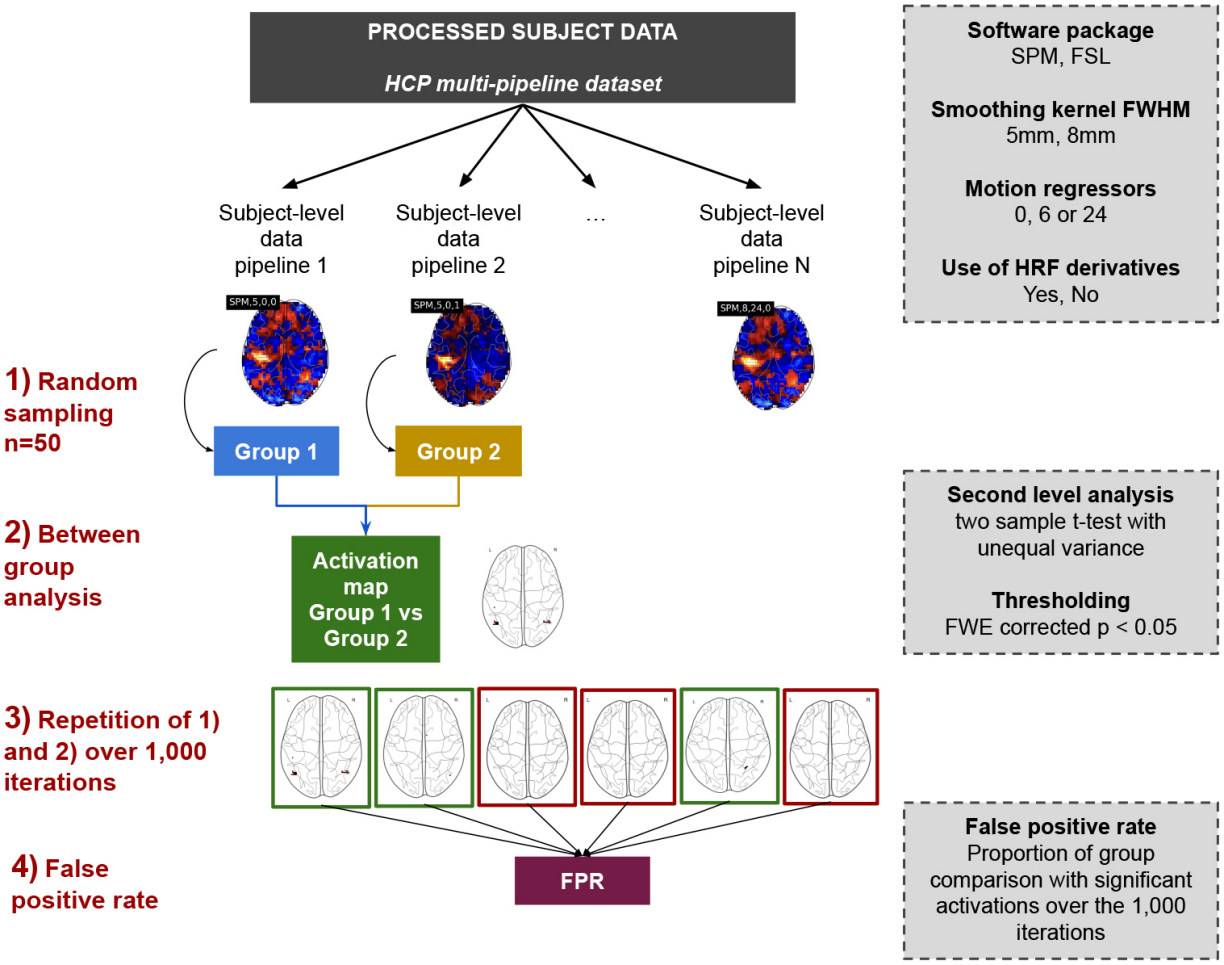
Overview of the method: (1) sampling of n = 50 subject-level contrast maps for each group (i.e., one group = one pipeline) from the HCP multi-pipeline, (2) between-group analyses “Group 1>or<Group 2”, (3) running 1,000 iterations of step 1 and step 2, and (4) estimation of the empirical rate of pipeline-induced significant differences referred to as the*false positive rate*.

All the scripts used to perform the study (group-level analysis and false positive rate estimation) are available on Software Heritage (Germani et al., 2025): swh:1:snp:585d3a0a3388a928ab3c6211c1826702aa618190.

### 2.1 HCP multi-pipeline

This study was performed using derived data from the HCP Young Adult (Van Essen et al., 2013). Written informed consent was obtained from participants, and the original study was approved by the Washington University Institutional Review Board. We agreed to the HCP Young Adult Open Access Data Use Terms available at: (“Human Connectome Project: Data Usage Agreement”, 2013).

Subject-level contrast and statistic maps from 1,080 subjects of the HCP Young Adult S1200 release ( Van Essen et al., 2013 ) for the motor task were obtained with 24 different pipelines. In brief, the pipelines implemented in the dataset varied on the following set of parameters:

- Software package: SPM (Statistical Parametric Mapping, RRID: SCR_007037) (Penny et al., 2006) or FSL (FMRIB Software Library, RRID: SCR_002823) (Jenkinson et al., 2012).
- Smoothing kernel: Full-Width at Half-Maximum (FWHM) of 5 mm or 8 mm.
- Number of motion regressors included in the General Linear Model (GLM) for the first-level analysis: 0, 6 (3 rotations, 3 translations), or 24 (3 rotations, 3 translations + 6 derivatives and the 12 corresponding squares).
- Presence (1) or absence (0) of the derivatives of the Hemodynamic Response Function (HRF) in the first-level GLM. The temporal derivative was added in FSL and both the temporal and dispersion derivatives in SPM.

These variations were chosen in particular due to the lack of consensus in the research community on their selection. In a many-analyst study (Botvinik-Nezer et al., 2020), 70 teams analyzed the same task-fMRI dataset using their usual pipeline, and final statistic maps were compared to explore potential differences between results across pipelines and the source of these differences. A complementary evaluation of the source of pipeline-based differences was also performed across three reproductions of published fMRI studies (Bowring et al., 2022). Within the varying parameters, smoothing kernel size, number of motion regressors, and design of the statistical analysis were all identified as impactful. Since there is a lack of consensus on the values of these parameters, they can easily vary from one pipeline to another. Therefore, shared derived data are likely obtained with pipelines for which these parameters are different.

In total, this led to 24 different subject-level pipelines (2 software packages×2 smoothing kernels×3 numbers of motion regressors×2 HRF). Together those contrast and statistic maps are referred to as the*HCP multi-pipeline dataset*. More details about the analysis and its implementation can be found inGermani et al. (2023). Briefly, the preprocessing steps included motion correction, coregistration of the functional images onto structural data, segmentation, registration into the MNI space, and smoothing. For subject-level statistical analysis, both SPM and FSL used a GLM to model experimental tasks, convolving event data with a Double Gamma HRF.

### 2.2 Between-group analyses

In this study, we explored the false positive rates induced by pipeline differences of between-group studies with subject-level contrast maps from different pipelines in three settings: within-pipeline (baseline), within-software (i.e., pipeline implemented in the same software package with different parameters), and between-software (i.e., pipeline implemented in different software packages with similar parameters).

#### 2.2.1 Contrast post-processing

As FSL and SPM use different MNI templates (Evans et al., 2012) (i.e., MNI152NLin6Sym for FSL, IXI549Space for SPM), subject-level contrast maps from different software packages had the same resolution (2 mm) but different dimensions. We used the following post-processing to harmonize the dimensions of the images. We used Nilearn (Abraham et al., 2014) (RRID: SCR_001362) to resample all subject-level contrast maps to the dimensions of the MNI152Asym2009 brain template with a 2 mm resolution using third-order spline interpolation (continuous interpolation in Nilearn, and the default parameter of the resampling function in the library). We masked the contrast maps using the intersection of all subject-level brain masks (all pipelines).

FSL and SPM contrast maps are also scaled differently (seeNichols, 2012). In both software packages, contrast maps are theoretically expressed in percent BOLD change but there are important differences in how this percent BOLD change is computed that effectively lead to scaling differences. Hence, in SPM, contrast maps units are closer to 2.5 times percent BOLD change due to the mask used to compute the global in-brain mean intensity. However, FSL contrast maps are scaled to 10,000 (i.e., 100 times percent BOLD change). We applied a factor to each contrast map to make them closer to percent BOLD change. Contrast maps in SPM and FSL were, therefore, rescaled by multiplying by100​/​250=0.4and100​/​10,000=0.01,respectively.

All between-group analyses were performed on resampled, masked, and re-scaled subject-level contrast maps. As a sanity check, we also computed the between-group same-pipeline analyses on the original contrast maps (i.e., before post-processing). As expected, the estimated false positive rates were consistent with the results obtained on post-processed data (seeSupplementary Table S1).

#### 2.2.2 Analysis setup

For each between-group analysis, we randomly sampled 100 participants without replacement among the full set of 1,080 participants and split them into 2 groups (N=50in each group). This sample size is larger than typical sample size in fMRI studies (around 30 participants) (Dockès et al., 2024)), but limiting atypical behaviors induced by small sample sizes. In each group, subject-level contrast maps were obtained using a different pipeline. This process was repeated for different groups and pairs of pipelines. We performed a one-tailed two-sample t-test with unequal variance and computed the statistic maps associated with$H_{0}$: “no mean difference of activation between groups.” We used a voxel-wisep<0.05FWE-corrected with Random Field Theory (Brett et al., 2004;Worsley et al., 1996), with approximately 130,000 comparisons (or 300–1,000 resolution elements (or “resels”), i.e., independent comparisons) per between-group analysis. All between-group analyses were performed in SPM in order to have consistent conditions in all the second-level analyses.

### 2.3 False positive rates

For a given pair of pipelines, the between-group analysis was repeated 1,000 times with different sets of participants. The empirical false positive rate was estimated as the proportion of between-group analyses, across the repetitions (seeSection 2) with at least one significant detection (seeFig. 1).

If the rate exceeds the nominalα-level of0.05(95% confidence interval[0.037;0.064]), we can conclude that pipeline-based differences impact the validity of results (toward invalidity), and if the rate is lower, we can conclude that the analysis is conservative. Of note, we use throughout the manuscript the term*validity*(respectively,*invalidity*) only in relation to type I error (i.e., specificity) as per the definition of this term in statistics. Measuring the effect of pipeline differences on type II error (i.e., sensitivity) is beyond the scope of the current manuscript.

### 2.4 Statistical distributions and P-P plots

P-P plots are usually used to observe how a given set of statistical values diverge from an expected distribution by plotting, for each$k^{th}$ordered statistical value, the expected associatedp-value on the x-axis and the obtainedp-value on the y-axis. Here, under the null hypothesis,p-values were expected to follow a uniform distributionU(0,1). Thus, for a set of N statistical values, the$k^{th}$orderedp-value follows a Beta distributionℬ(k,N−k+1)with expected valuek​/​(N+1)(David & Nagaraja, 2003;Ge et al., 2012). Confidence bounds on thep-values were computed using the Beta distribution.

Here, we used a Bland–Altman ( Giavarina, 2015 ) variant of P-P plots. Bland–Altman plots provide a visual representation of the difference between two measurements on the y-axis and the average of the two measurements on the x-axis. Here we adapted those plots to p -values, as follows:

- on the x-axis: the expectedp-value in$-log_{10}$
- on the y-axis: the difference between the$-log_{10}$obtained and the$-log_{10}$expectedp-values.

This update made it easier to observe the behavior in the tails of thep-value distribution (which is of interest here). High statistical values (right tail of our sample) are associated with lowp-values, that is, to high$-log_{10}$p-values. We also looked at the distributions of the statistical values for multiple between-group analyses, and compared them with a Student distribution$T_{98}$.

## 3 Results

### 3.1 Analyses using the same pipeline (baseline)

Table 1shows the false positive rates obtained for all analyses with the same pipeline in both groups, separately for SPM and FSL. For all combinations, the false positive rates were below the expected value of 0.05, ranging between 0.012 and 0.028 for SPM and between 0.013 and 0.024 for FSL. These results, obtained with the same pipeline in both groups, are used as a baseline in the following.

**Table 1.** False positive rates for between-groups analyses with the same pipeline in both groups, with SPM and FSL and for all possible sets of parameters (number of motion regressors, smoothing kernel FWHM, and presence or absence of HRF temporal derivatives).

	**SPM**			
	Smooth 5 mm		Smooth 8 mm	
	No derivatives	Derivatives	No derivatives	Derivatives
0 motion regressors	0.012	0.013	0.016	0.023
6 motion regressors	0.015	0.006	0.024	0.013
24 motion regressors	0.023	0.016	0.025	0.028

	**FSL**			
	Smooth 5 mm		Smooth 8 mm	
	No derivatives	Derivatives	No derivatives	Derivatives
0 motion regressors	0.014	0.013	0.015	0.023
6 motion regressors	0.018	0.014	0.018	0.018
24 motion regressors	0.015	0.013	0.016	0.024

The rates were always under 0.05.

### 3.2 Analyses using pipelines with different parameters

**Fig. 2.**
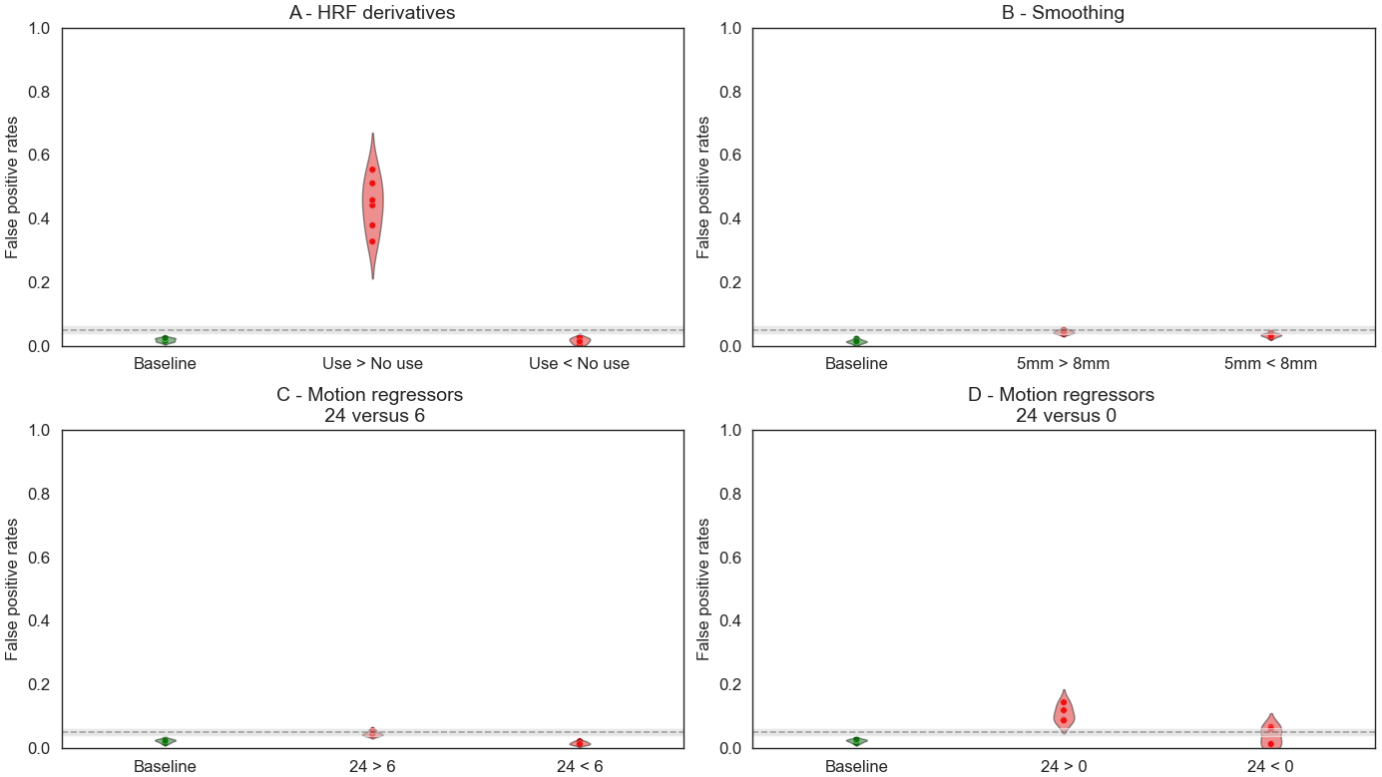
False positive rates for pipelines with a single differing parameter in SPM: (A) HRF derivatives, (B) smoothing, and (C) and (D) motion regressors. For each, we provide the false positive rates obtained for 1/ Baseline analysis with default parameters, used as a reference (Green, first column) and 2/ Default>Variation and Default<Variation (Red, second and third columns). The gray dashed line corresponds to the alpha level (0.05), and the gray band to the corresponding confidence interval at 95%.

The following subsections present the results obtained with pipelines using different set of parameters (within software). In each case, we looked at the false positive rate (Fig. 2), the statistical distributions (Supplementary Figures S3 and S11), and the associated P-P plots (Figs. 4and5;Supplementary Figures S6 and S9). To present the results, we chose a default value for each studied parameter—smoothing 5 mm FWHM, HRF with derivatives, and 24 motion regressors—and compared our results with those obtained with the default.

#### 3.2.1 Different HRF

Adding derivatives to model the HRF was the most impactful of all three varying factors in both software packages. The false positive rates obtained with different HRF (i.e.,*canonical HRF*>or<*HRF with derivatives*) are presented inFigure 2Afor the six analyses performed (i.e., with varying levels of smoothing and number of motion regressors—with the same setting in both pipelines).

In SPM, the comparison*canonical HRF*>*HRF with derivatives*(Fig. 2A) showed invalid false positive rates (above the theoretical 0.05 threshold) for all pipeline combinations. Similarly, in FSL, all combinations gave invalid results for this same comparison except two combinations:*5mm*or*8mm smoothing FWHM*and*24 motion regressors*. These two analyses led to values that were within the confidence bounds of the 0.05 threshold or slightly conservative (0.032 and 0.061, respectively). For the opposite comparison (i.e.,*canonical HRF*<*HRF with derivatives*), all combinations resulted in valid results with false positive rates under 0.05.

Figure 4andSupplementary Figure S6show the corresponding Bland–Altman P-P plots for comparisons with different HRF and otherwise default parameters. In both software packages, consistently with what we observed for the false positive rates, the comparison*canonical HRF*>*HRF with derivatives*led to values that were outside of the 95% confidence interval (gray area). In SPM, values were further away from the 95% confidence interval than in FSL.

The same observations could be made on the statistical distributions for both SPM and FSL (Supplementary Figures S3 and S11): both showed a shift in mean and variance, but this was smaller for FSL. The combination of pipeline parameters used in this Figure (i.e., pipelines with*5mm*FWHM and*24 motion regressors*, with different HRF derivatives) showed nearly valid false positive rates, as stated in the previous paragraph (seeFig. 2), which could explain why the shift seemed smaller in FSL compared with SPM. We also observed the P-P plots for a different combination of FSL pipelines with other parameters (5 mm, 0 motion regressors) inSupplementary Figure S8and found a similar shift as the one observed for SPM.

#### 3.2.2 Different smoothing

The false positive rates obtained with different levels of smoothing (*5mm*or*8mm*) in the pipelines are presented inFigure 2Bfor the 6 analyses performed (i.e., with varying HRF models and number of motion regressors—with the same setting in both pipelines).

The false positive rates obtained with different levels of smoothing (*5mm*>or<*8mm*) in the pipelines were above the 0.05 theoretical rate in FSL (ranging from 0.07 to 0.16) and within the confidence interval around the theoretical rate in SPM (ranging from 0.03 to 0.05). Compared with the baseline analyses using the same pipelines, the false positive rates were always inflated and were slightly higher for the tail*5mm*>*8mm*.

The Bland–Altman P-P plots (Fig. 4;Supplementary Figure S6) are consistent with the observations made on the false positive rates. Between-group analyses using pipelines with different smoothing gave results outside of the 95% confidence interval in FSL and within the interval in SPM, with only a small positive difference in the direction*5mm*>*8mm*.

The behaviors observed on the P-P plots can be explained by the positive shift in mean values and standard deviations observed on the statistical distribution for*5mm*>*8mm*for FSL (Supplementary Figure S11), which is less pronounced for SPM (Supplementary Figure S3).

#### 3.2.3 Different number of motion regressors

The false positive rates obtained with different number of motion regressors (0, 6, and 24) are presented inFigure 2C and Dfor the 6 analyses performed (i.e., with varying levels of smoothing and different HRF—with the same setting in both pipelines). We studied the combinations*24 motion regressors*>or<*6 motion regressors*(third column) and*24 motion regressors*>or<*0 motion regressors*(fourth column).

In SPM, false positive rates were below the 0.05 theoretical rate for all comparisons of*24 motion regressors*>or<*6 motion regressors*. For the comparison with no motion regressors, the false positive rates were higher and above 0.05 for*24 motion regressors*>*0 motion regressors*and slightly below for the opposite. In FSL, the results were dependent on the other pipeline parameters. All combinations led to invalid results (i.e., above the theoretical 0.05 threshold) except for*24 motion regressors*>*0/6 motion regressors*when using the canonical HRF (i.e., no HRF derivatives) in both pipelines.

InSection 3.2.1, we showed that all combinations of pipelines with varying HRF models led to invalid results except those with*5mm*or*8mm smoothing*and*24 motion regressors*. Here, we also observe invalid results for all combinations of pipelines with*24 motion regressors*>or<*0/6 motion regressors*, except those with*5mm*or*8mm smoothing*and*no HRF derivatives*. We can suppose that in FSL, when using*24 motion regressors*, the use of HRF derivatives in the GLM has a low impact on the results and similarly, when using the*canonical HRF*, using*0, 6, or 24 motion regressors*does not change the results much, and thus has a low impact on the validity of the mega-analyses combining subject-level data obtained from pipelines with different parameters.

In the Bland–Altman P-P plot for SPM (Fig. 4), we observed more extreme values in the P-P plots for the comparisons “*24 motions regressors*>or<*0 motion regressors*” than for those of “*24 motions regressors*>or<*6 motion regressors,*” which is consistent with our observations on the false positive rates. The Bland–Altman P-P plot (Supplementary Figure S6) for FSL with 5 mm smoothing and an HRF with derivatives, the comparison*24 motion regressors*>or<*0 motion regressors*were consistent with the invalid false positive rates found with such parameters: we found conservative results for the comparison*24 motion regressors*>*0 motion regressors*(plain line) and invalid results in the opposite direction (dashed line).

Statistical distributions (Supplementary Figures S3 and S11) also show a shift in mean and variance for the comparison “*24 motion regressors*>or<*0 motion regressors,*” for both SPM and FSL. This shift is not as important for the comparison “*24 motion regressors*>or<*6 motion regressors.*” The comparison “*6 motion regressors*>or<*0 motion regressors*” was also showed for comparison, and showed similar results as the “*24 motion regressors*>or<*0 motion regressors*” comparison.

#### 3.2.4 Combined effects of parameters

We observed the combined effects of

- differences in smoothing and in HRF model
- differences in smoothing and in motion regressors.

The false positive rates obtained with different smoothing and different HRF models or different motion regressors in the pipelines are presented inFigure 3for the different analyses performed.

**Fig. 3.**
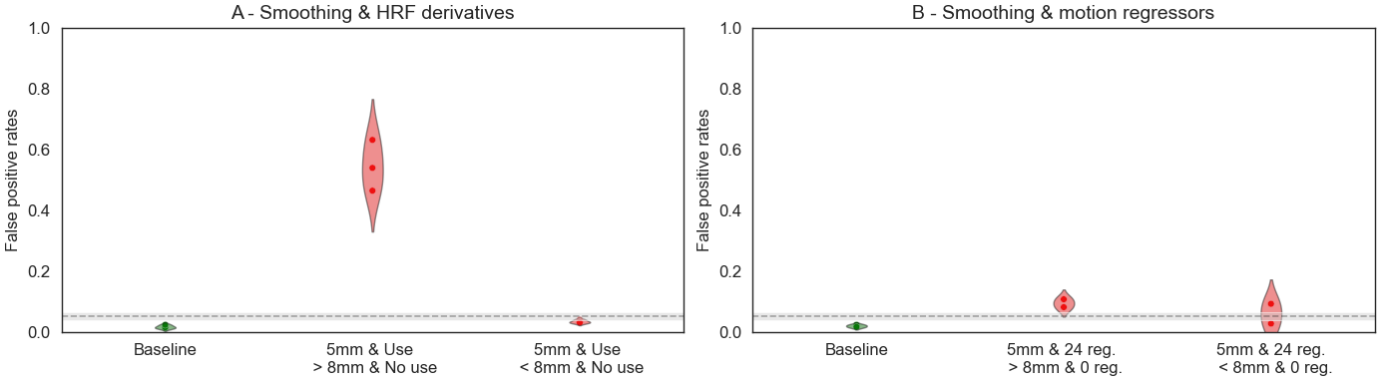
False positive rates for pipelines with two differing parameters in SPM: (A) Smoothing and HRF, (B) Smoothing and motion regressors. For each studied parameter, we provide the rates obtained for 1/ Baseline analysis with default parameters, used as a reference (Green, first column) and 2/ Default>Variation and Default<Variation (Red, second and third columns). The gray dashed line corresponds to the alpha level (0.05) and the gray band to the corresponding confidence interval at 95%.

**Fig. 4.**
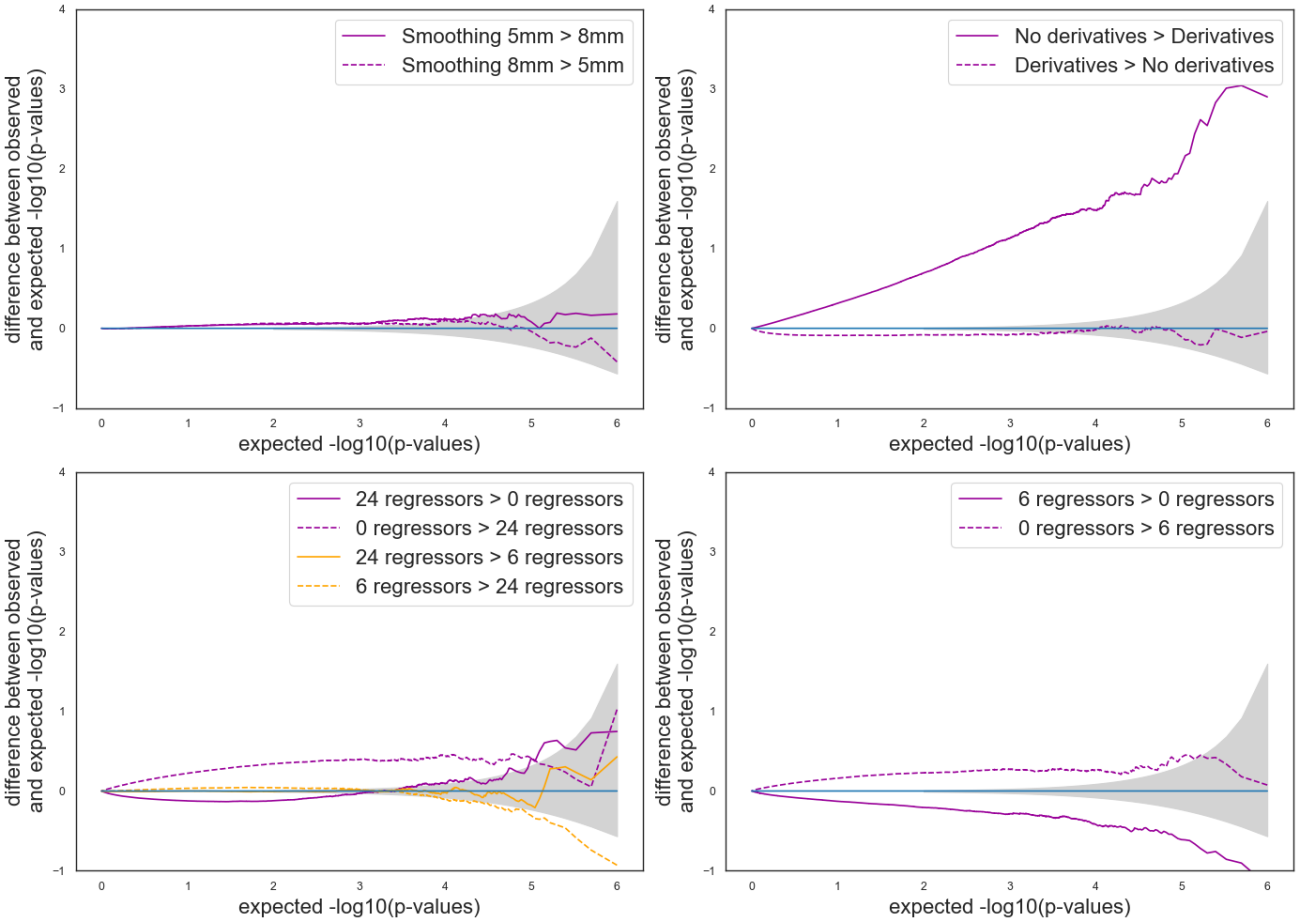
Bland–Altman P-P plots for pipelines with a single differing parameter in SPM. The gray shade corresponds to the 0.95 confidence interval. A curve above (respectively, below) the confidence interval indicates invalidity (respectively, conservativeness). Default parameters: 5 mm smoothing, 24 motion regressors, and no HRF derivatives.

In both SPM and FSL, the first set of between-group analyses (*5mm smoothing*,*canonical HRF*)>(*8mm smoothing*,*HRF with derivatives*) led to invalid results with false positive rates largely above the 0.05 theoretical threshold (around 0.60). The opposite test provided conservative results.

In SPM, the results for (*5mm smoothing*,*canonical HRF*)>(*8mm smoothing*,*HRF with derivatives*) were close to those obtained for the analyses with a single varying parameter*canonical HRF*>*HRF with derivatives*(from 0.46 to 0.63 in the combined effect analysis and from 0.32 to 0.52 in the exploration of HRF derivatives effect only, seeFig. 2A). In the isolated analyses, the effect of changing the smoothing kernel FWHM was not very important in SPM (“5 mm vs 8 mm smoothing kernel FWHM”), which might explain why the false positive rates did not increase much in the combined effect analyses.

Under FSL, the previous analyses on the effect of each of these parameters separately (changing smoothing kernel FWHM and changing HRF model separately) both gave inflated false positive rates, and their combined effect largely increased the false positive rates (up to 0.77) compared with the effect of changing the use HRF derivatives alone (up to 0.49).

Similar observations can be made on the P-P plots inFigure 5andSupplementary Figure S9.

**Fig. 5.**
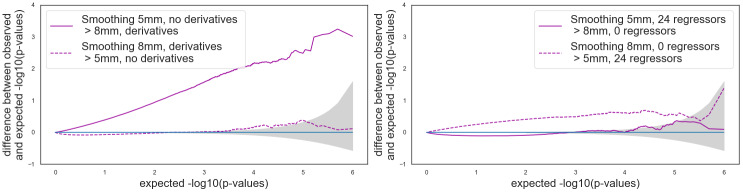
Bland–Altman P-P plots for pipelines with two differing parameters in SPM. The gray shade corresponds to the 0.95 confidence interval. A curve above (respectively, below) the confidence interval indicates invalidity (respectively, conservativeness). Default parameters: 5 mm smoothing, 24 motion regressors, and no HRF derivatives.

In both SPM and FSL, the second set of analyses (*5mm smoothing*,*24 motion regressors*)>or<(*8mm smoothing*,*0 motion regressors*), we found invalid results for nearly all combinations. In SPM, the false positive rates were only slightly above the theoretical threshold of 0.05 (0.081 and 0.11), which is consistent with our previous observation: initially, changing smoothing kernel FWHM and number of motion regressors separately led to false positive rates close to 0.05, consistently, their combination led to rates that were only slightly invalid.

For both SPM and FSL, we observed shifts in the distributions of statistical values (Supplementary Figures S3 and S11). These shifts were similar to those obtained for changes in motion regressors only.

### 3.3 Analyses using pipelines with different software packages

We also explored the ability to use in the same between-group analysis subject-level data obtained with different software packages (here FSL and SPM). We performed the analyses for all possible combinations SPM>or<FSL: 2 smoothing kernels×3 numbers of motion regressors×2 HRF models, corresponding to 12 between-software comparisons—with the same setting for both SPM and FSL pipelines. The false positive rates are displayed inFigure 6. For all between-software analyses, the false positive rates were above 0.05. We obtained lower values for*SPM*>*FSL*(between 0.10 and 0.32), than for the opposite test (between 0.56 and 0.95). In all cases, false positive rates were largely increased compared with the reference analyses (i.e., using the same software in both groups). This observation was consistent with the P-P plot, which showed a large deviation from the 95% confidence interval for the direction*SPM*<*FSL*(Fig. 7).Figure 8shows the distribution of statistical values for the between-software comparison with all other parameters set with default values (i.e., 5 mm smoothing kernel, 24 motion regressors, and no HRF derivatives). We can see a shift in terms of mean and standard deviation of values. This shift was larger than those observed, for instance, for the effect of HRF derivatives, which was the most impacting factor on within-software comparisons.

**Fig. 6.**
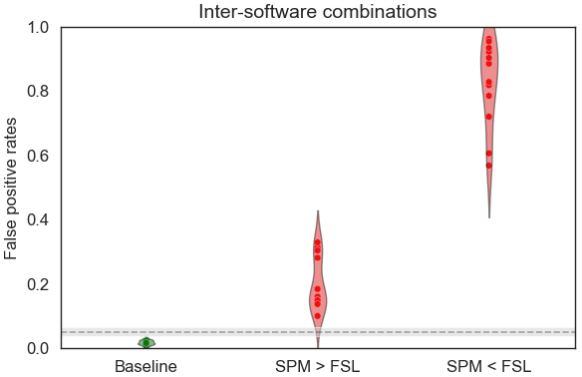
False positive rates for pipelines with different software packages. We provide the false positive rates obtained for 1/ the corresponding analyses within-pipelines, that is, the baseline (Green, first column) and 2/ analyses for “FSL>SPM” and “FSL<SPM” (Red, second and third columns). The gray dashed line corresponds to the alpha level (0.05) and gray band to the corresponding confidence interval at 95%.

**Fig. 7.**
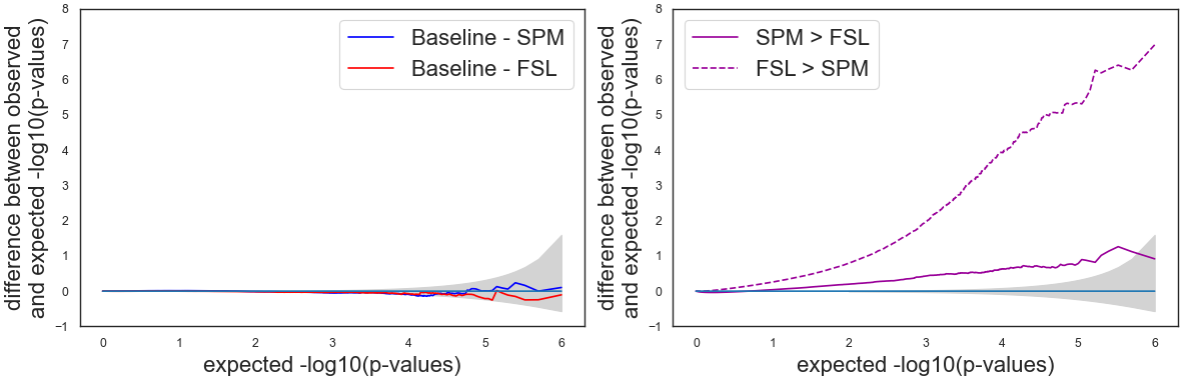
Bland–Altman P-P plots for pipelines with different software packages. The gray shade corresponds to the 0.95 confidence interval. A curve above (respectively, below) the confidence interval indicates invalidity (respectively, conservativeness). Default parameters: 5 mm smoothing, 24 motion regressors, and no HRF derivatives. Note that for these two plots, the y-axis range has been modified to (-1, 8) to adapt to the particularly high false positive rates given by these combinations of pipelines.

**Fig. 8.**
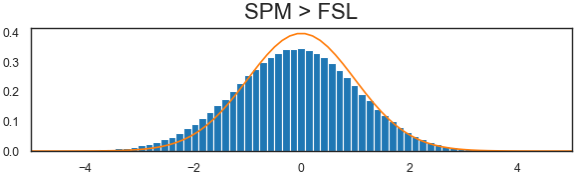
Distribution of statistical values for between-software analyses, compared with the expected distribution.

## 4 Discussions

In this study, we showed that between-group analyses that use data generated by different pipelines can lead to invalidity when differences in pipelines are not properly accounted for. In almost all cases, combining data processed with different pipelines led to false positive rates above the theoretical 0.05 threshold. These results, obtained when combining subject-level contrast maps processed differently, suggest that it is necessary to consider how analytical variability may affect the results when combining data.

When performing analyses using the same pipeline on all participants’ data (as traditionally done in the literature), results were valid for all analyses. Although the false positive rates obtained were lower than the 5% rate, the results were similar to those obtained inEklund et al. (2016). The level of smoothing, combined with the thresholding method that we chose (i.e., voxel-wise FWE-corrected based on random field theory in SPM), may be responsible for these lower rates (Nichols & Hayasaka, 2003).

Our results for different pipeline analyses suggest that some factors have a larger impact than others. We saw that for differences regarding the size of the smoothing kernel and number of motion regressors (6>or<24 motion regressors) within SPM software package, results were similar to those obtained with identical pipeline analyses, suggesting that participant data can be combined without having to consider the differences in pipelines, if this is the only difference. This is not the case for differences in the use of HRF derivatives and use of motion regressors (0 motion regressors>or<6 or 24 motion regressors), which gave invalid results.

We also saw that combining multiple differences in parameters could result in bigger effects, depending on the effect of each parameter alone. The combination of two parameters that both have a high effect on the results led in our case to inflated false positive rates, while the combination of parameters that had a limited effect did not lead to higher false positive rates (e.g., smoothing and motion regressors in SPM). This suggests that it may be possible to model the effect caused by specific variations in the subject-level pipelines. To enable this in the future, it is essential that the pipelines used is shared with enough details to allow a reproduction of the exact processing applied on the data.

However, the ability to model the effect of parameters is limited to specific variations. For example, for each variation of parameter, we saw different effects across the two software packages under study (SPM and FSL). Overall, observations were similar, but false positive rates were often increased in FSL compared with SPM for the same comparison. This suggests that some parameter values are more robust to changes when combined together, here, in FSL, when using 24 motion regressors, combining data with different use of HRF derivatives led to false positive rates close to the baseline analysis (i.e., same pipeline in both groups).

The most important source of invalidity was found when studying the effect of differences in software packages. SPM and FSL both implement similar pipeline steps with different settings. While we tried to align some parameters between the two software packages by changing the software package default values (e.g., smoothing kernel, type of HRF, etc.), some steps are specific to each software and cannot be changed by the user, causing potential differences between the results. We tried to correct some of these differences, in particular for the unit scale of subject-level contrast maps. But, even with these corrections, we still found highly inflated false positive rates when comparing pipelines with the same values for the parameters under study and different software packages. We suppose that differences in how software packages scale the data were not compensated by our simple rescaling approach and that more work will be needed to be able to combine subject-level data from two different software packages in the same analysis.

In this study, we focused on between-group analyses in which each group of participants was processed with a different pipeline. While this an extreme setup (in which the effect of interest is perfectly confounded with differences in pipelines), in practice, other combinations may be observed, for example, with multiple pipelines used within a group. Our setup—in which processing pipelines varied depending on the group—was justified by the use-case in which data from various public datasets are used in the same analysis. For example, specific datasets have been created to study various neurological disorders, usually associated with a minimal processing pipeline dedicated to the study, and the corresponding minimally processed data (Alzheimer’s Disease Neuroimaging Initiative (ADNI) (Jack Jr. et al., 2008) for Alzheimer’s disease, Autism Brain Imaging Data Exchange (ABIDE) (Di Martino et al., 2014) for autism, etc.). Researchers may want to use these minimally processed data and compare groups of participants with one group composed of participants with specific conditions from one of these processed datasets, and the other group composed of healthy participants from another processed dataset (e.g., from the Human Connectome Project;Van Essen et al., 2013).

We chose to study variations induced by four types of parameters (software package, HRF, smoothing, and number of motion regressors), within each software package based on their widespread use in the neuroimaging community (Carp, 2012). Yet, in practice, there are many more variations: researchers might use different software versions, perform or not specific sub-steps in the analysis (for example, the use or not of slice-timing correction), use different HRF models, etc. Therefore, in real conditions, the differences observed between pipelines will likely be more important. In future work, other analyses may be done for other varying parameters using the same framework.

For other types of confounds such as imaging site, scanner effect, or age and sex, harmonization or mitigation methods have been proposed to take these into account. Several studies (Alfaro-Almagro et al., 2021;Beer et al., 2020;Fortin et al., 2016;Rao et al., 2017) proposed to remove confounds by incorporating them as additional regressors in the analysis, or by estimating batch-specific parameters (such as mean and variance) and then using these to standardize the data with frameworks such as ComBat (Fortin et al., 2016). Alternative approaches have also been proposed, such as restriction, where the study is limited to participants with specific characteristics. InZarnani et al. (2019), authors employed this method in a cohort study focused on males of the same age and nationality. In practice, pipeline-based differences may be accounted for by adding a confound in the statistical analysis (see for instance;Alfaro-Almagro et al., 2021), or using harmonization techniques such as ComBat (Fortin et al., 2016). However, the specific impact of pipeline-based differences remained unexplored. Our results can be used to understand in which cases differences in analytical pipelines must be accounted for.

Recently, deep learning frameworks, and in particular generative models used for style transfer (Gatys et al., 2016), showed their potential for such task in converting data between different domains (e.g., acquisition site) (Liu et al., 2021;Nguyen et al., 2018). Currently, the most widespread practice is to include a covariate in the statistical model for pipeline-based confounds. We envision that other methods such as style transfer may provide additional solutions to mitigate analytical variability in such analyses (see for instance;Germani, Maumet, et al., 2024).

## 5 Conclusion

Our study shows that between-group analysis using subject-level data which have been processed differently can be affected by pipeline-based differences. While some parameters did not have significant effects, others produced invalid results, suggesting that it is necessary to model those pipeline-based confounds.

## Supplementary Material

Supplementary Material

## Data Availability

This study was performed using derived data from the HCP Young Adult (Van Essen et al., 2013), publicly available at ConnectomeDB. Data usage requires registration and agreement to the HCP Young Adult Open Access Data Use Terms available at: (“Human Connectome Project: Data Usage Agreement”, 2013). The HCP multi-pipeline dataset (Germani et al., 2023) is available on Public-nEUro (“Public nEUro”, 2023) athttps://doi.org/10.70883/GTKK1541. **Software environment**All analysis pipelines were executed in Python v3.8. The executions require the installation of SPM and FSL software packages. To facilitate reproducibility, we provide a NeuroDocker image that can be pulled from Dockerhub and that contains all necessary software packages. The Docker image is available at:https://hub.docker.com/r/elodiegermani/open_pipeline. **HCP Multi-pipeline**Python scripts to run the pipelines and create the dataset were made available publicly in the Software Heritage public archive: swh:1:dir:67ce4a985abc2206169943486b91db7acb998a54 (Germani, Fromont, et al., 2024). **Between-group analyses, figures, and tables** Python and Matlab scripts to run the experiments and to create the figures and tables of this article are available in the Software Heritage public archive: swh:1:snp:27d3bec940e435478942c9085f5a1410763827da ( Germani et al., 2025 ).
Programming language: Python3.8, Matlab License: MIT Requirements: multiple Python libraries, available in the Docker container open_pipeline
