# Supplementary material for: On the validity of fMRI mega-analyses using data processed with different pipelines
Source: Imaging Neurosci (Camb). 2025 Apr 28;3:imag_a_00522. doi: 10.1162/imag_a_00522 (PMC12319730; doi:10.1162/imag_a_00522)
Supplement: Supplementary Material [file imag_a_00522-supp.pdf]

Supplementary materials

Supplementary methods

Bland-Altman P-P plots For a given pair of pipelines, we have 1,000 group analyses, which makes a total of more than 150M voxel values. Since the resulting list of voxels obtained is very large and using it for further observations can be very time-consuming, for each between-group analysis using two given pipelines, on which we wanted to make observations, we only took a random sample of 1,000,000 values from the concatenation of statistical values over the 1,000 corresponding group analyses.

To compute the p-values, we transformed the statistic values using the survival function of the Student's t-distribution with 98 degrees of freedom (50 participants + 50 participants - 2). This corresponds to 1-CDF (cumulative distribution function) of the t-distribution. The confidence intervals were computed using a beta distribution for each kth value with the lower bound being k and the upper bound being 1, 000, 000 - k + 1. After conversion to logarithmic scale, this gave us the confidence intervals for the distribution of p-values.

Supplementary table

SPM

	Smooth 5 mm		Smooth 8 mm	
	No derivatives	Derivatives	No derivatives	Derivatives
0 motion regressors	0.014	0.019	0.025	0.019
6 motion regressors	0.013	0.015	0.021	0.025
24 motion regressors	0.021	0.015	0.018	0.019

FSL

	Smooth 5 mm		Smooth 8 mm	
	No derivatives	Derivatives	No derivatives	Derivatives
No motion regressors	0.01	0.013	0.014	0.014
6 motion regressors	0.015	0.017	0.017	0.022
24 motion regressors	0.017	0.02	0.014	0.012

Supplementary Table 1: False positive rates for between-groups analyses with the same pipeline in both groups, using contrast maps without post-processing with SPM and FSL and for all possible sets of parameters (number of motion regressors, smoothing kernel FWHM and presence or absence of HRF temporal derivatives). The rates were always under 0.05.

Supplementary figures for analyses within SPM

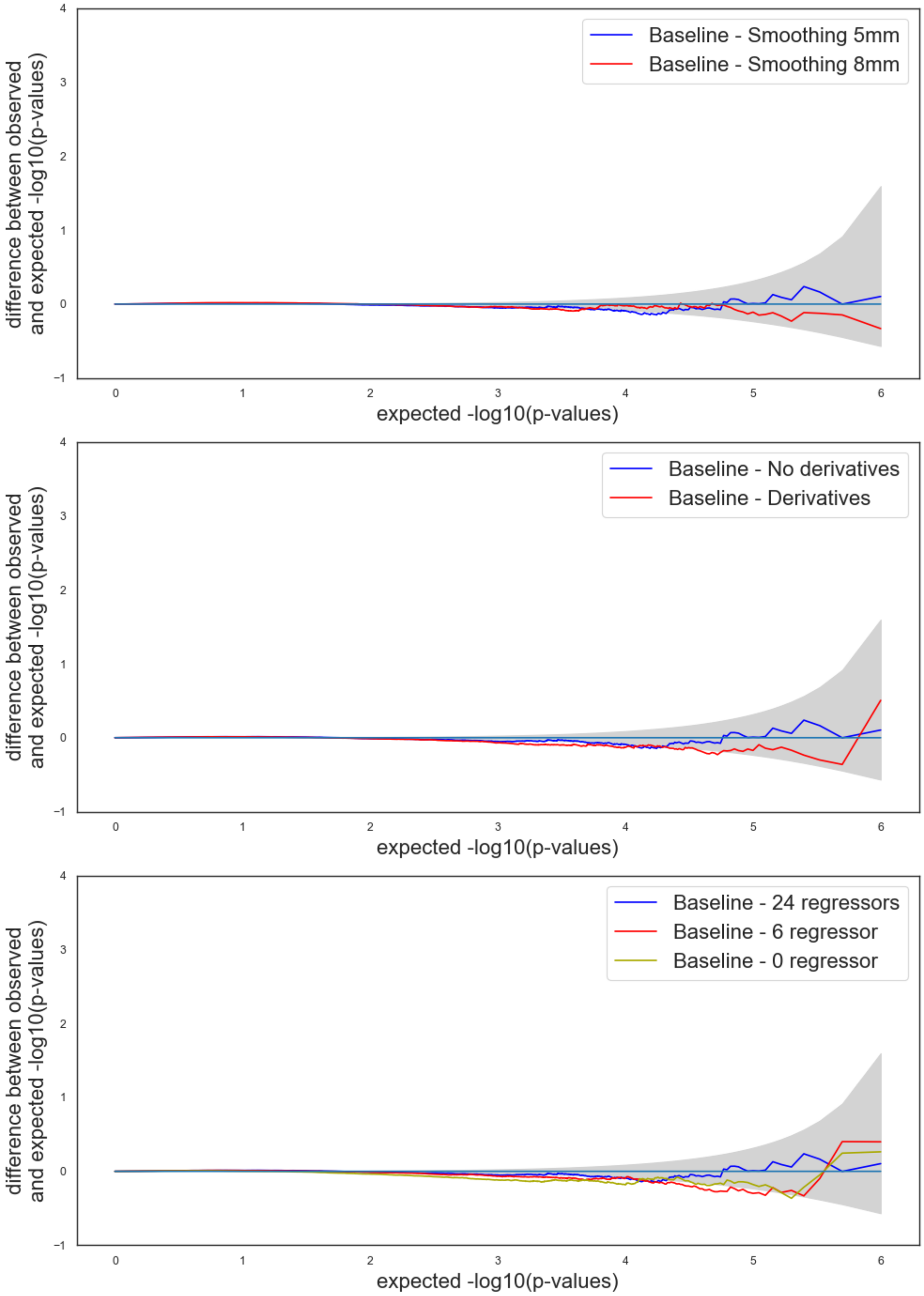

Supplementary Figure 1: Baseline for Figure 4. Bland-Altman P-P plots for pipelines with no differing parameters in SPM. The grey shade corresponds to the 0.95 confidence interval. A curve above (respectively below) the confidence interval indicates invalidity (respectively conservativeness). Default parameters: 5 mm smoothing, 24 motion regressors and no HRF derivatives.

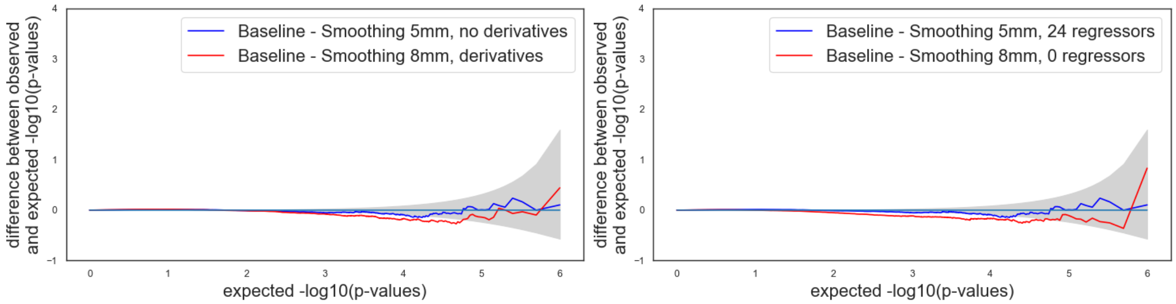

Supplementary Figure 2: Baseline for Figure 5. Bland-Altman P-P plots for pipelines with no differing parameters within SPM. The grey shade corresponds to the 0.95 confidence interval. A curve above (respectively below) the confidence interval indicates invalidity (respectively conservativeness). Default parameters: 5 mm smoothing, 24 motion regressors and no HRF derivatives.

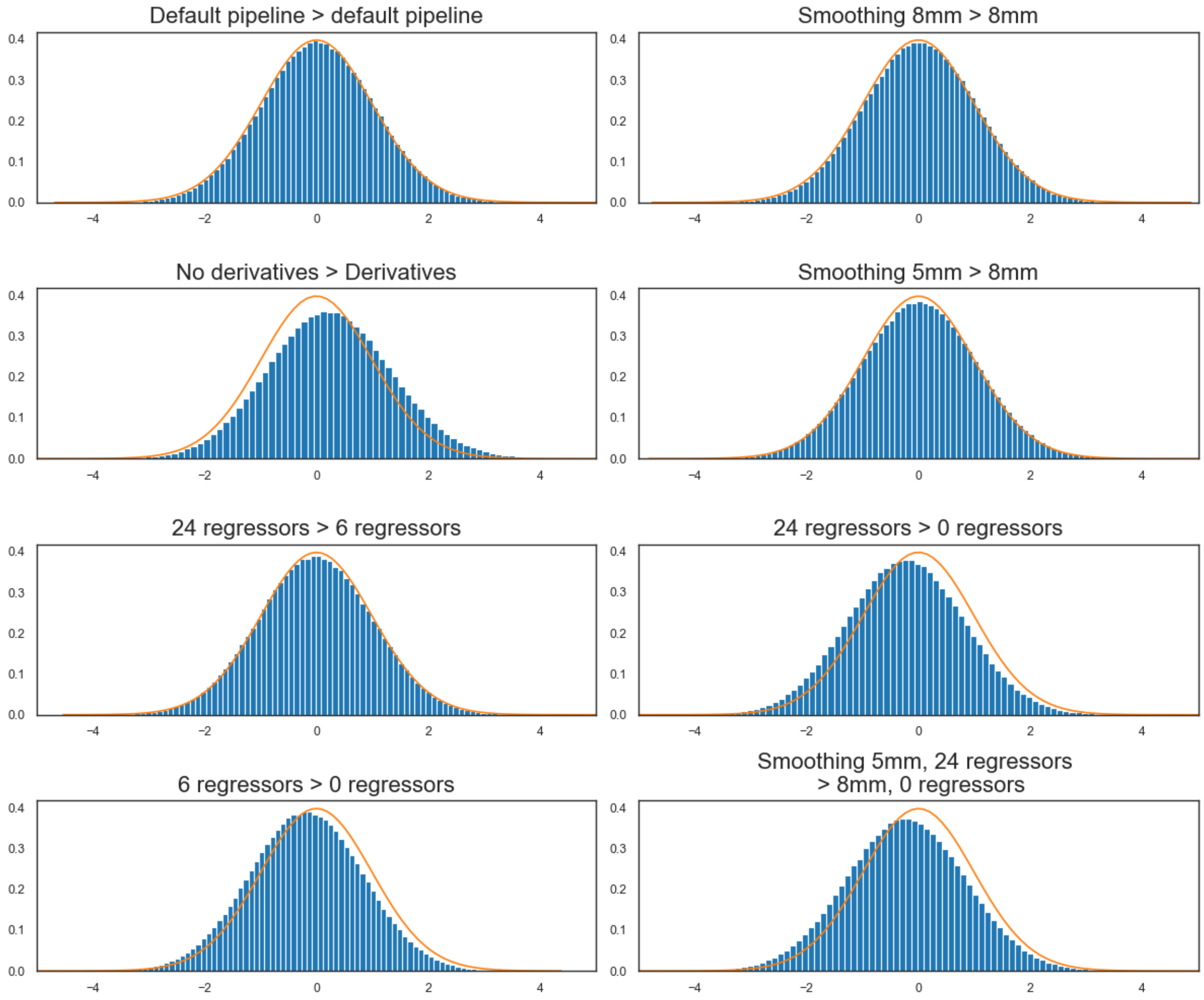

Supplementary Figure 3: Distribution of statistical values for multiple between-group analyses under SPM, compared to the expected distribution. Default parameters: 5 mm smoothing, 24 motion regressors and no HRF derivatives. Pipelines which differ from the default pipeline are put in bold. The orange curve represents the Student distribution with 98 degrees of freedom, which is the expected distribution in our case under null hypothesis.

Supplementary figures for analyses within FSL

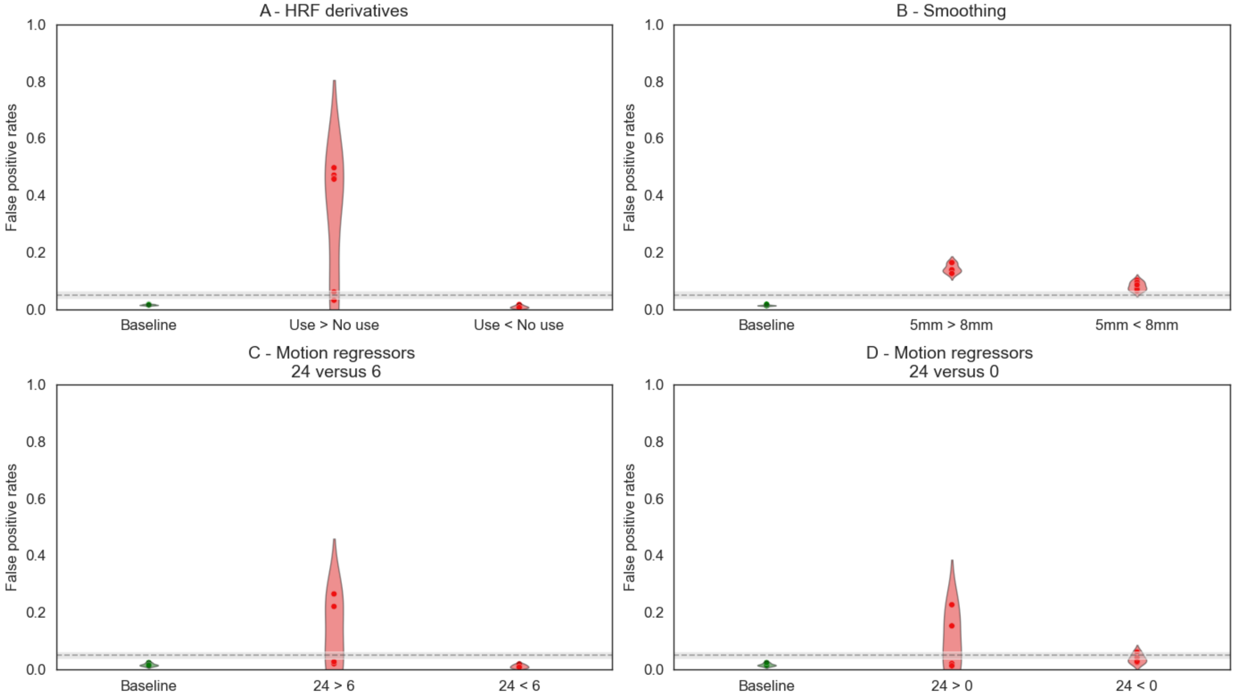

Supplementary Figure 4: False positive rates for pipelines with a single differing parameter in FSL: A) HRF derivatives, B) smoothing and C and D) motion regressors. For each, we provide the false positive rates obtained for: 1/ Baseline analysis with default parameters, used as a reference (Green, first column) and 2/ Default > Variation and Default < Variation (Red). The grey dashed line corresponds to the alpha level (0.05), and the grey band to the corresponding confidence interval at 95%.

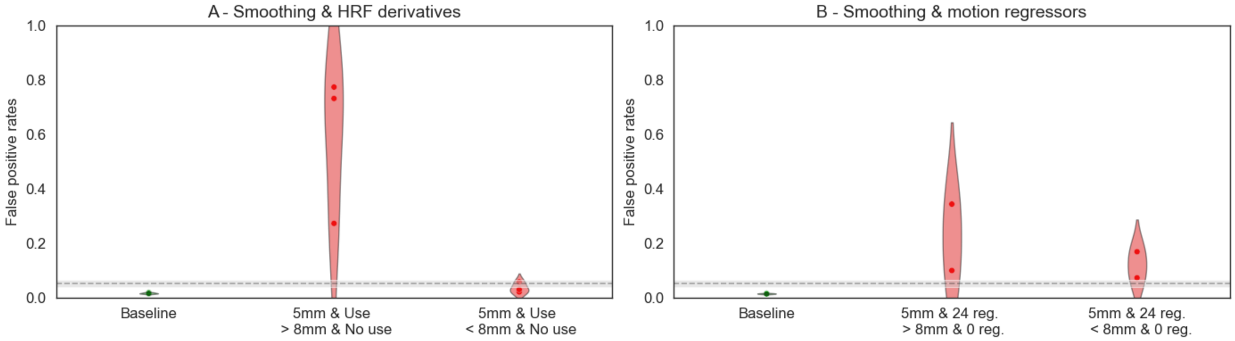

Supplementary Figure 5: False positive rates for pipelines with two differing parameters in FSL: A) Smoothing and HRF, B) Smoothing and motion regressors. For each studied parameter, we provide the rates obtained for: 1/ Baseline analysis with default parameters, used as a reference (Green, first column) and 2/ Default > Variation and Default < Variation (Red). The grey dashed line corresponds to the alpha level (0.05) and grey band to the corresponding confidence interval at 95%.

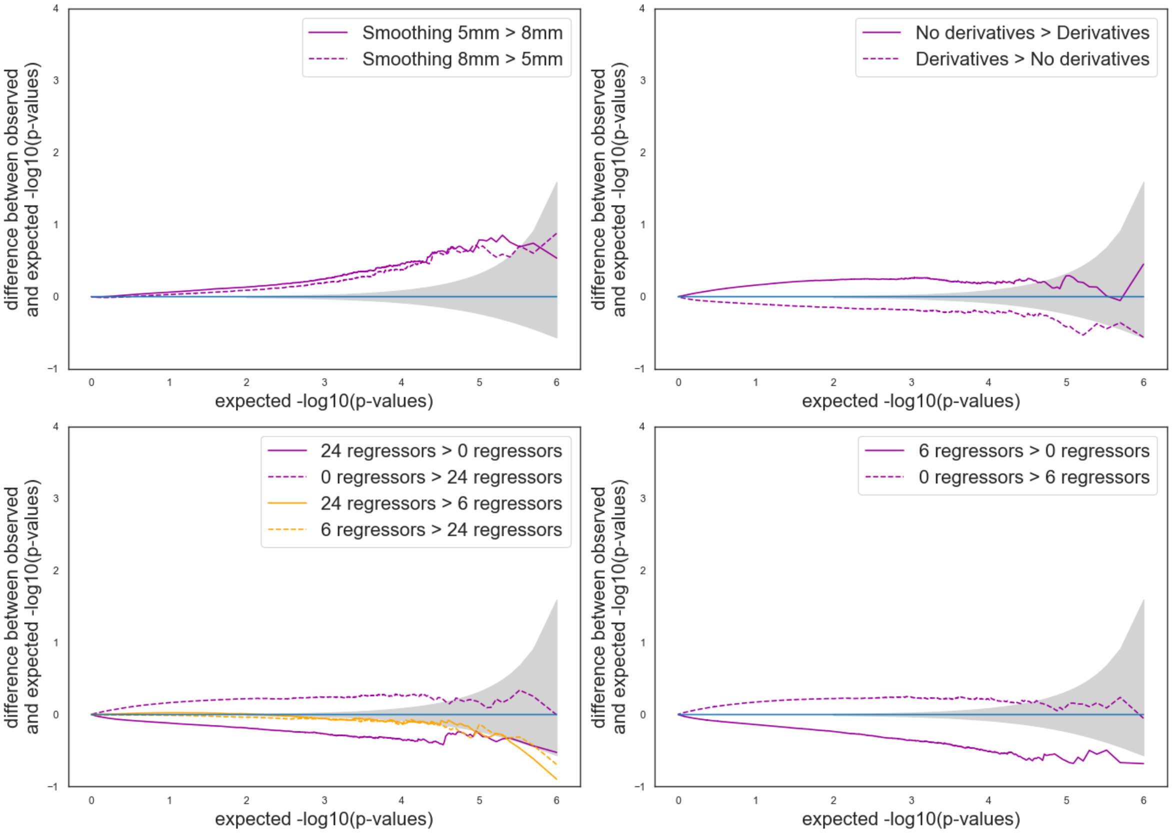

Supplementary Figure 6: Bland-Altman P-P plots for pipelines with a single differing parameter in FSL. The grey shade corresponds to the 0.95 confidence interval. A curve above (respectively below) the confidence interval indicates invalidity (respectively conservativeness). Default parameters: 5 mm smoothing, 24 motion regressors and no HRF derivatives.

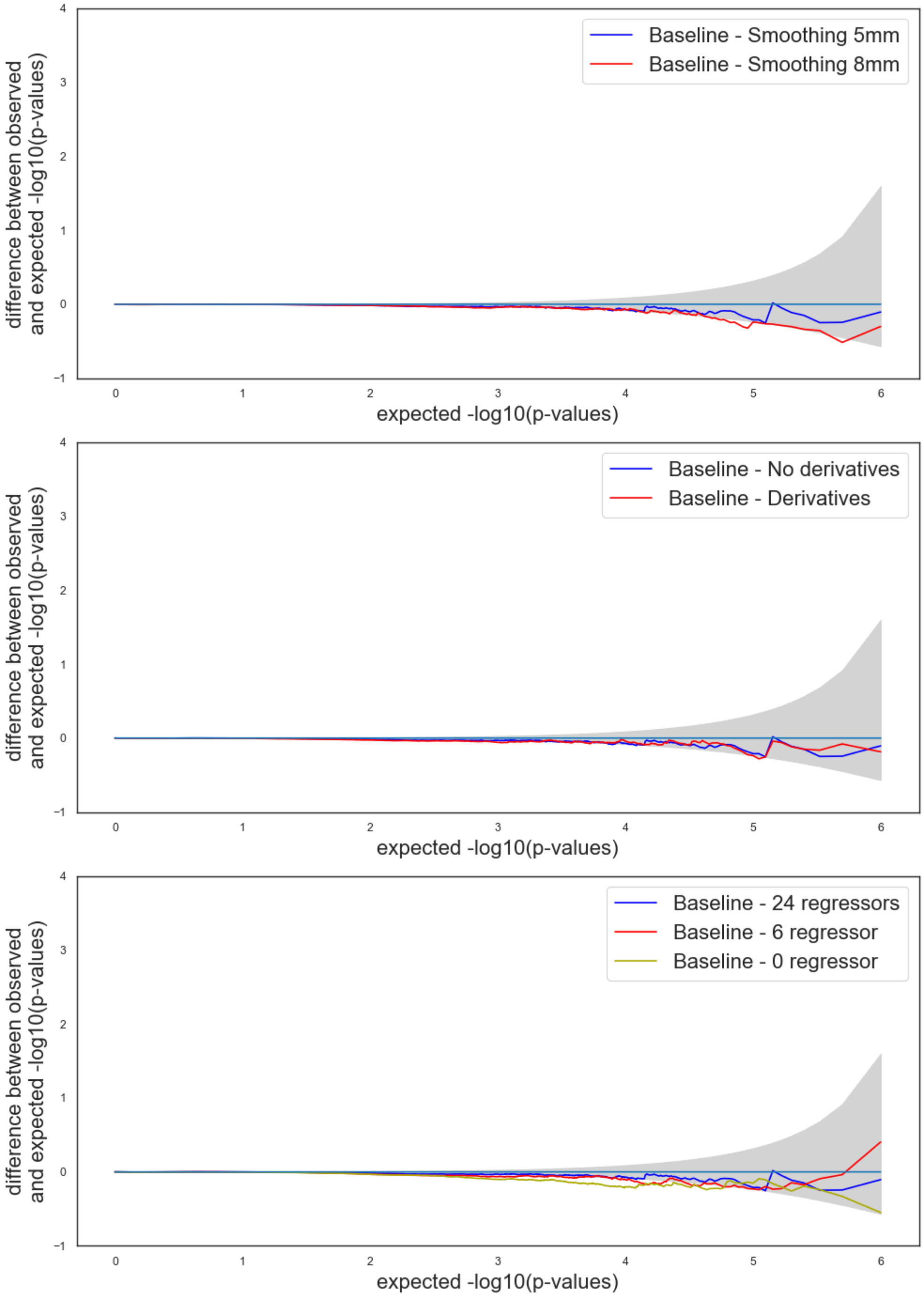

Supplementary Figure 7: Baseline for Supplementary Figure 6. Bland-Altman P-P plots for pipelines with no differing parameters in FSL. The grey shade corresponds to the 0.95 confidence interval. A curve above (respectively below) the confidence interval indicates invalidity (respectively conservativeness). Default parameters: 5 mm smoothing, 24 motion regressors and no HRF derivatives.

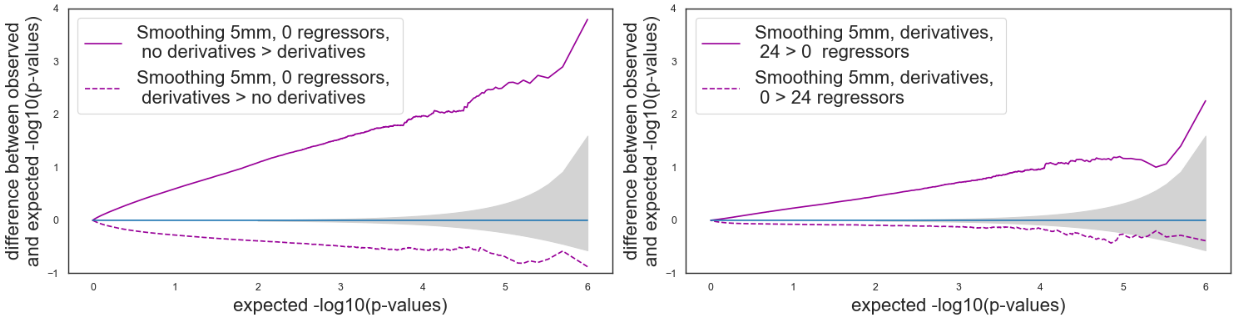

Supplementary Figure 8: Bland-Altman P-P plots for pipelines with a single differing parameter in SPM. The grey shade corresponds to the 0.95 confidence interval. A curve above (respectively below) the confidence interval indicates invalidity (respectively conservativeness). Default parameters values were modified to 5 mm smoothing, 0 motion regressors and no HRF derivatives to explore the impact of fixed parameters on the validity of analyses.

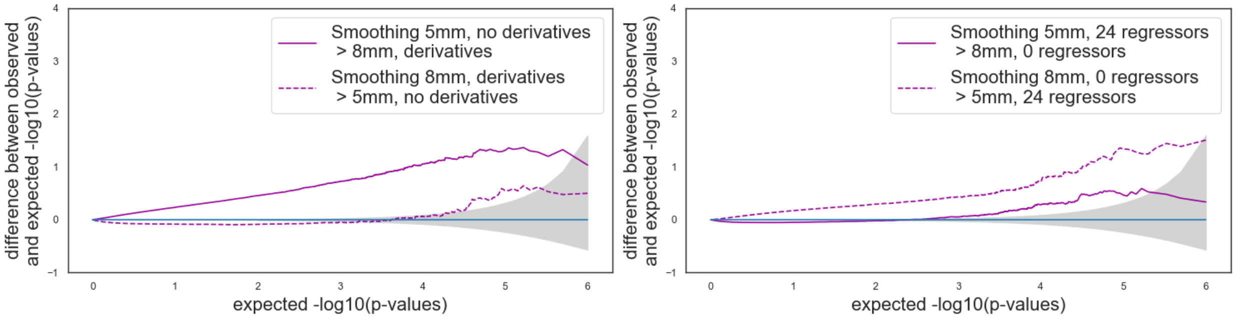

Supplementary Figure 9: Bland-Altman P-P plots for pipelines with two differing parameters in FSL. The grey shade corresponds to the 0.95 confidence interval. A curve above (respectively below) the confidence interval indicates invalidity (respectively conservativeness). Default parameters: 5 mm smoothing, 24 motion regressors and no HRF derivatives.

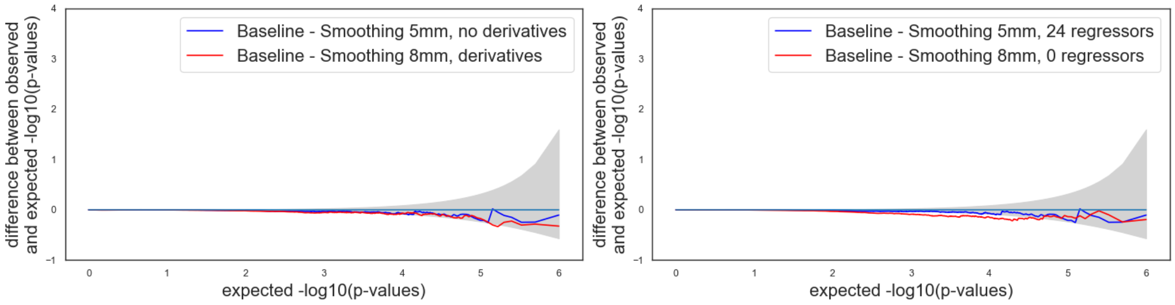

Supplementary Figure 10: Baseline for Figure 9. Bland-Altman P-P plots for pipelines with no differing parameters in SPM. The grey shade corresponds to the 0.95 confidence interval. A curve above (respectively below) the confidence interval indicates invalidity (respectively conservativeness). Default parameters: 5 mm smoothing, 24 motion regressors and no HRF derivatives.

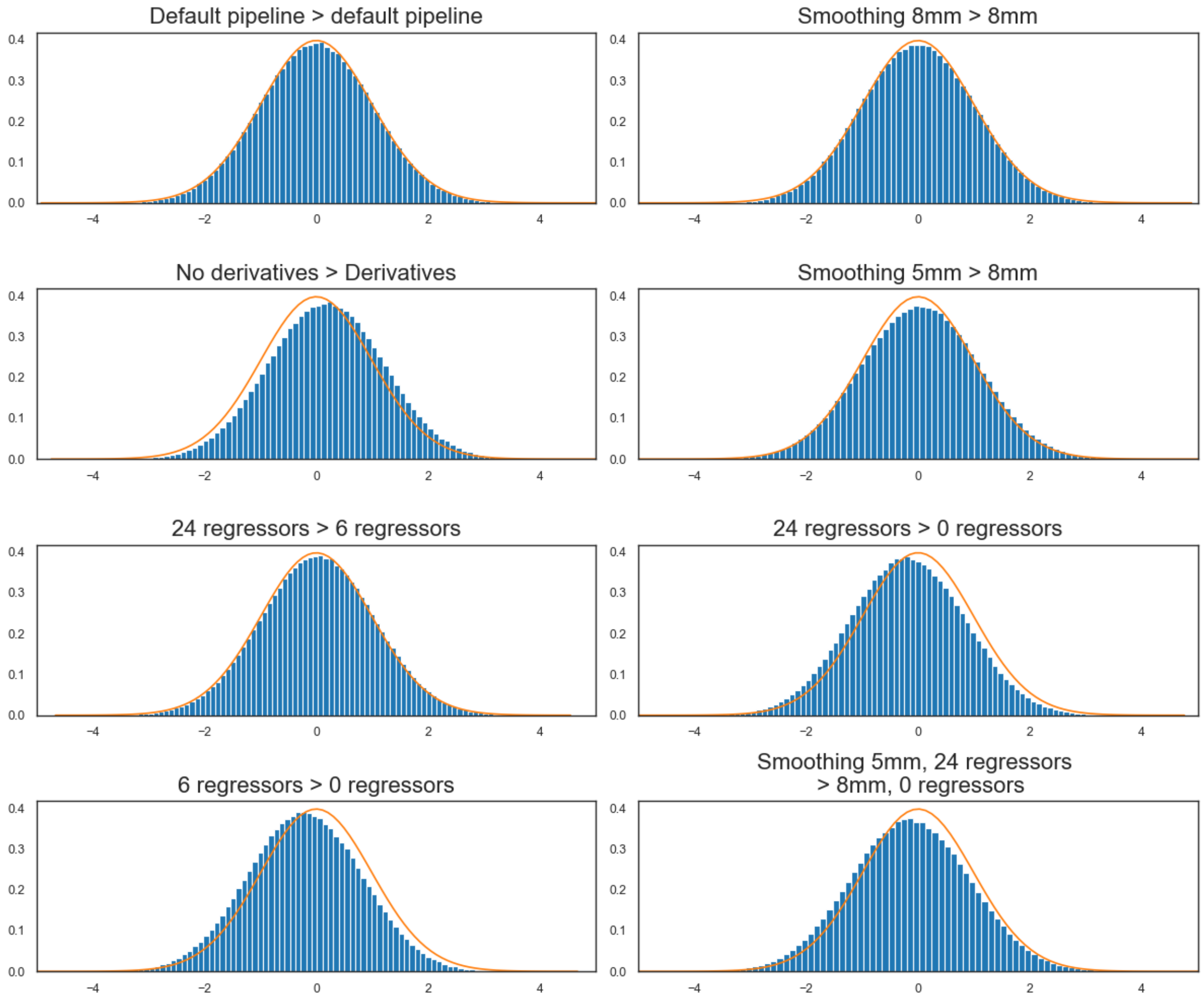

Supplementary Figure 11: Distribution of statistical values for multiple between-group analyses under FSL, compared to the expected distribution. Default parameters: 5 mm smoothing, 24 motion regressors and no HRF derivatives. Pipelines which differ from the default pipeline are put in bold. The orange curve represents the Student distribution with 98 degrees of freedom, which is the expected distribution in our case under null hypothesis.
